# Anxiety in adolescence. Can we prevent it?

**DOI:** 10.4317/medoral.21754

**Published:** 2016-12-06

**Authors:** Anna Llorca, Elisabeth Malonda, Paula Samper

**Affiliations:** 1 Basic Psycology Department, University of Valencia, Avda. Blasco Ibáñez, 21, 46010 Valencia, Spain

## Abstract

**Background:**

Emotions are potent modulators and motivators of the behaviour that the individual displays in the different situations they have to live and they can act as a protection factor or vulnerability of the adapted or maladaptive behaviour. This study focuses on anxiety in adolescence. Objectives. The objective is, through a longitudinal study, to analyse the psychological processes and emotions that facilitate the symptoms of anxiety and those which protect the adolescent from these symptoms.

**Material and Methods:**

417 adolescents (192 boys and 225 girls) participated in a three-wave longitudinal study in Valencia, Spain. In the first wave, adolescents were either in the third year of secondary school (81 boys and 85 girls) or the fourth year of secondary school (111 boys and 140 girls). The mean age was 14.70 (SD = 0.68; range = 13-17 years). This study monitored participating adolescents for three years.

**Results:**

The results indicate a differential profile in the evaluated emotions according to sex, with the girls being the ones to experiment more anxiety and more empathy, while the boys show more emotional instability and aggression.

**Conclusions:**

It is concluded that the best predictors for anxiety are anger state, aggressive behaviour, empathic concern together with the lack of coping mechanisms focused on problem solving and the perception of stress as a threat.

**Key words:**Adolescence, anxiety, emotions, coping, stress.

## Introduction

In the last decades, the study of emotions and their importance in the behaviour of the individual has reached a central interest in research. The ability to know one’s emotions and perceive the emotions of others makes interpersonal relationships easier. In the same way, self-regulation of negative emotion, the capacity of self-control when facing situations that cause tension, together with the inhibition of impulsiveness, make it easier to reach effective resolution of conflict and adapted behaviour. In short, emotions are potent modulators and motivators of the behaviour that the individual displays in the different situations they have to live and they can act as a protection factor or vulnerability of the adapted or maladaptive behaviours.

The study of emotions is of special importance in adolescence, stage in which important changes are introduced at all levels: biological changes, psychological changes, maturity changes, all of them teamed with the importance of the group, the peers and the making of decisions at a personal, vocational or professional level. Stage in which has been verified an increase of impulsiveness correlated to more aggressive behaviour in interpersonal relationships and even to more crime (1,2).

Anxiety disorders have a high prevalence in adolescence and this prevalence rises if anxiety symptoms (3), are taken into consideration, said symptoms, have important physiological manifestations (breathing difficulties, dry mouth, shaking hands, tachycardia), as well as psychological manifestation (excessive worry, fear without reason, panic states) and they have a negative influence in how the adolescent functions, in their academic performance, the acceptance by their peers, the aggressive behaviour and depression.

Anxiety considered as a negative emotional arousal state, often accompanied by a concern about a potential future threat that results in distress (4), highlights the importance of emotional regulation in maintaining anxiety symptoms.

A recent revision of studies in anxiety in childhood and adolescence shows that deficits in emotional competence, empathy, emotional self-efficacy, ability to cope in situations that cause tension and generate negative emotions are processes that could be related to anxiety symptoms (3).

In the same way, some studies conclude the relation between depression and emotions and uncontrolled conducts like aggressive behaviour, anxiety, anger, emotional instability o personal discomfort (5).

Some authors have pointed out that the ability to discriminate and understand emotions, as well as the lack or decreased empathy has influence over depression and anxiety (6). The emotional changes, the mood changes are related to what is happening to us, our experiences, therefore, certain situations can take us to depression and anxiety.

In the present study we consider analysing the relation between different representative variables of emotional competence and anxiety, with a longitudinal study, throughout adolescence. The objective is to establish the variables with higher predictor power of anxiety symptoms and those that discriminate between more or less anxious adolescents. It is of special interest to differentiate between the cognitive processes and emotions that facilitate anxiety symptoms and those that protect the adolescent from said symptoms.

Moreover, focusing on adolescence the gender variable is included to analyse if a different emotional profile exists between boys and girls.

## Material and Methods

- Participants

417 adolescents participated in a three-wave longitudinal study in Valencia, Spain. The sample consisted of 192 boys and 225 girls. In the first wave, adolescents were either in the third year of secondary school (81 boys and 85 girls) or the fourth year of secondary school (111 boys and 140 girls). The mean age was 14.70 (SD = 0.68; range = 13–17 years). This study monitored participating adolescents for three years.

Most adolescents came from two-parent households where parents were married (83.7% married; 13.2% divorced). In terms of educational attainment, 21.8% of mothers had less than a secondary school diploma, 42.2% had a secondary school diploma or equivalent and 30.7% had some university education. Similarly, 24% of fathers had less than a high school diploma, 41% had a high school diploma or equivalent and 28.7% had some university education. Most students self-identified themselves as being from Spain (86.6%). Small percentages of the remaining students self-identified themselves as being from Latin America (e.g., 3.4% from Ecuador, 2% from Colombia and 1.1% from Bolivia) and Eastern European countries (e.g., 1.7% from Romania). Participating schools were randomly selected from the list of all schools in Valencia with students enrolled in compulsory secondary education. In total, 11 schools participated in the study.

- Procedure

The research project and the evaluation proposal were presented to the school management team to ask for their authorisation and cooperation.

Approval from the School Council and parental consent were obtained. Participation by students was voluntary; students were free to decline to participate. The survey was administered by trained researchers in the classroom in 50-minute sessions during school hours. The annual assessments took place in three successive years during the first trimester of the school year. The study followed all ethical guidelines, respecting respondents’ anonymity for both data collection and data analysis.

- Measures

Anxiety and Stress Scales (DASS) (7). Evaluates anxiety symptoms and stress during the last week. Example item ‘I realized my mouth was dry’. Cronbach’s alpha for this study was .81 at time 1, .84 at time 2 and .85 at time 3).

Physical and Verbal Aggression Scale (AFV) (8,9). This instrument uses 20 items to evaluate behaviours that harm others physically or verbally. Example item: ‘I fight’, ‘I threaten my classmates’. Cronbach’s alpha for this study was .80 at time 1, .82 at time 2 and .83 at time 3).

Emotional Instability Scale (EI) (8,9). It describes the behaviour that indicates a lack of self-control in social situations as a result of the scarce ability to curb impulsiveness and emotionality. Example item: ‘I am impatient’, ‘I interrupt others when they talk’. Cronbach’s alpha for this study was .82 at time 1, .79 at time 2 and .83 at time 3).

State and Trait Anger Scale (STAXI-N) (10). It evaluates the anger as a state. Example of item: ‘I am furious’, ‘I want to fight’. Cronbach’s alpha for this study was.76 at time 1; α = .88 at time 2; α = .90 at time 3). It evaluates anger trait. Example of item: ‘I get easily irritated’, ‘I am grumpy’ (α = .74 at time 1; α = .76 at time 2; α = .77 at time 3).

Stress Appraisal Measure for Adolescents (SAMA), adapted from Stress Appraisal Measure (11). Evaluates how people interpret stressing events. The participants have to answer items about the way they usually think and feel when they face a stressing situation. The evaluated factors are stress as a challenge (example item: ‘I can face positively the situations that cause me stress’, ‘I have what I need to face stress’) and stress as a threat (example item: ‘Stress has a negative impact on me’, ‘I feel anxious’). Cronbach’s alpha for this study was .79 (challenge) and .79 (threat) at time 1, .82 (challenge) and .81 (threat) at time 2 and .84 (challenge) and .84 (threat) at time 3.

The Interpersonality Reactivity Index (IRI) (12), captures participants’ empathy levels. It measures cognitive (fantasy empathy and perspective taking) and emotional (empathic concern and personal distress) dimensions of empathy. We used emotional dimensions for this study. The empathic concern scale refers to feelings of compassion, concern, and care when seeing others in distress. An example item is “I often have feelings of tenderness and concern towards those less fortunate than I am.” The personal distress scale captures feelings of anxiety and distress evoked by others’ negative experiences. An example item is “In dire or emergency situations I feel uncomfortable.” The reliability of this instrument, measured by Cronbach’s alpha, is as follows: personal distress (.60), empathic concern (.60) at time 1, personal distress (61), empathic concern (66) at time 2, and personal distress (65) and empathic concern (64) at time 3.

Adolescent Coping Scale (13,14). Evaluates how adolescents cope with their problems in general. In particular, 18 coping strategies are differentiated: Seek social support, Focus on solving the problem, Making an effort and be successful, Worry, Invest in close friends, Seek belonging, Get their hopes up, The strategy of lack of coping, Tension reduction, Social action, Ignore the problem, Self-blame, Keep it to oneself, Seek spiritual support, Focus on the positive, Seek professional help, Seek relaxing diversions and Physical distraction. These strategies can be classified in three basic coping styles: coping focused on problem solving (example item: ‘I focus on solving what is causing the problem’), coping by seeking support in the relationship with others (example item: ‘talk to others to know what they would do if they had the same problem’) and unproductive coping (example item: ‘cry or shout’). The reliability of this instrument, measured by Cronbach’s alpha, is as follows: problem-solving (74), support-relationship-others (84), unproductive (83) at time 1; problem-solving (76), support-relationship-others (84), unproductive (86) at time 2, and problem-solving (79), support-relationship-others (83), unproductive (87) at time 3.

- Data analyses

First of all, a repeated measurements mix variance analysis has been done taking as a factor among the subjects the sex variable to analyse if there are differences between boys and girls taking part during the three evaluated waves for each of the evaluated variables (anxiety, perspective taking, empathic concern, physical and verbal aggression, anger trait and state, emotional instability, coping strategies and stress). On the other hand, we have carried out an analysis of hierarchical regression being this variable dependent on the anxiety that adolescents manifest at time three of the evaluation, with the objective to conclude the evaluated variables with higher predictor power over it in the previous two times, and analyse if the cognitive variables or the emotional variables evaluated are more compelling. The varied regression analysis technique has allowed us to summarise the research findings with the construction of a predictor profile of the variables included in the investigation. The reliability of the psychometric instruments has been measured trough Cronbach’s alpha.

## Results

 Table 1 represents the typical measurements and deviations of the emotional and coping variables evaluated throughout the time and by sex.

**Table 1:**
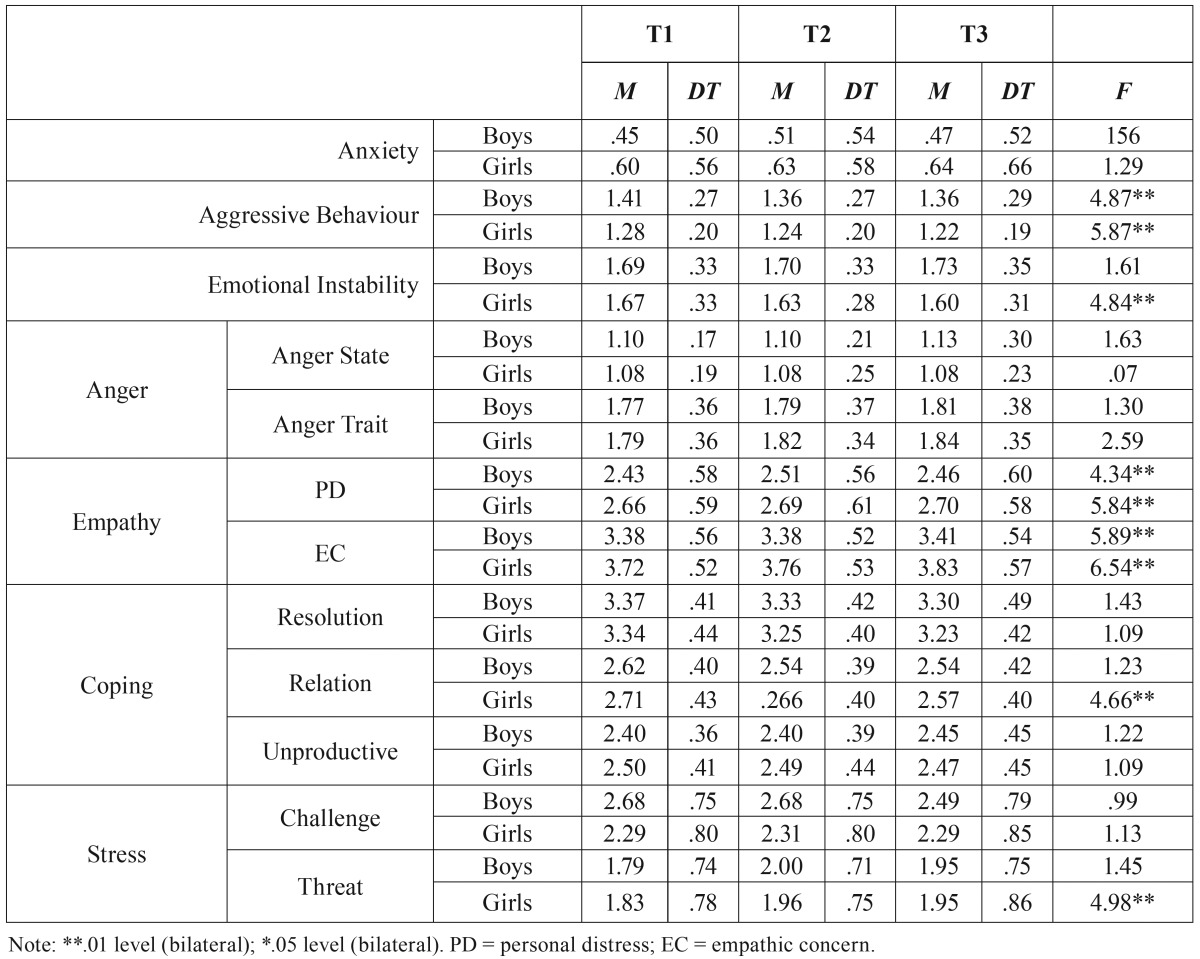
Typical measurements and deviations. Evolution of the emotional and coping variables evaluated by sex throughout the 3 years.

The analysis of repeated measurements show that there aren’t relevant differences in anxiety throughout the time and that the interaction effect between the sex variable and the anxiety variable is not significant, which indicates to us that there isn’t a different evolution profile of anxiety in boys and girls. However, there are differences between boys and girls in the evaluated time 2 and time 3, being adolescent girls those who score higher in anxiety. Physical and verbal aggressive behaviour diminishes throughout time significantly in boys and girls. Likewise, the boys score even higher in the three evaluated times.

As for emotional instability, while in the boys it rises slightly in throughout the time, in the girls it diminishes significantly. In time 2 and 3, the boys score significantly higher than the girls in emotional instability. In regards to empathy, the differences are significant in both emotional subscales depending on the sex variable, scoring the girls higher in both subscales throughout the three evaluated times.

There aren’t significant differences in anger state-trait throughout the time or due to sex in any of the evaluated times.

With regards to the coping variable evaluated in its three dimensions (focused on problem solving, in the relationship with others and the unproductive one), the results show that, first of all, the strategies focused on problem solving diminish significantly in both girls and boys but there aren’t any differences in relation to sex. As for the strategies focused on the relationship with others, the trend is the same, meaning it diminishes significantly in boys and girls but the interaction in this case is significant. Both in time 1 and time 2 the adolescent girls score higher in this coping dimension and in relation to unproductive coping, there aren’t significant differences throughout time nor due to sex in any of the evaluated times.

Finally, in relation to the variable of stress perceived as a threat, it rises significantly in time 2 to diminish in time 3, but the differences aren’t significant in relation to sex, whilst perceived as a challenge, it also diminishes in time 3, scoring the boys higher than the girls in a significant manner.

The hierarchical regression analyses have been carried out taking the adolescents anxiety in time 3 as the criteria variable. The first block of the equation includes the emotional variables (emotional instability, ager state and trait, aggressive behaviour, empathy) evaluated in time 1; the second block includes the emotional variables evaluated in time 2; the third block includes the coping and stress variables evaluated in time 1; and finally, the fourth block adds the coping and stress variables evaluated in time 2. The multiple colinear tests have been satisfactory, with all inflation factors of variance of less than 2.00 and the tolerance of all variable close to 1.00.

 Table 2 shows the summary of the hierarchical regression analysis of the emotional, coping and stress variables in the adolescents. Due to the relatively big size of sample, which results in an increase of the power, α was fixed as *p* ≤ 0.01.

**Table 2:**
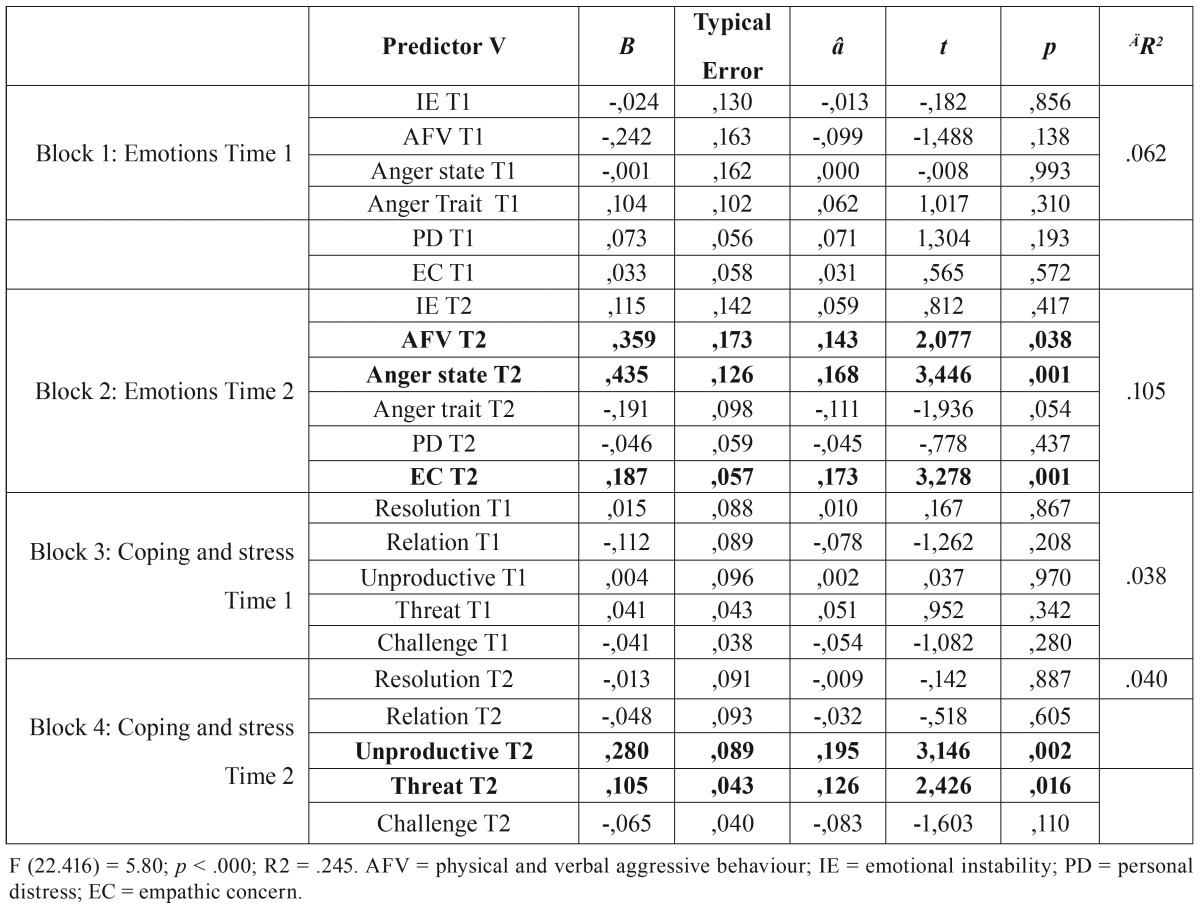
Summary of the regression analysis per block of emotional and stress variables in times 1 and 2 in anxiety evaluated in time 3.

The global prediction for anxiety, has been significant (F(22.416) = 5.80; *p* < .000) (see  Table 2).

The complete model explains the 24.5% of the variance in anxiety of the adolescents of both sexes when they are between 15 and 18 years of age, with the emotional variables evaluated in time 1 explaining the 16.6% of the variance in block 1. The emotional variables evaluated in time 2 represent an additional 10.5% of the variance in block 2. The coping and stress variables evaluated in time 1 represent an additional 3.8% of the variance in block 3 while the coping and stress variables evaluated in time 2 represent the remaining 4% variance in block 4. The emotional as well as the coping and stress variables evaluated in time 1 have no predictor power in the anxiety perceived and felt by the adolescents of both sexes when the are between 15 and 18 years of age. On the other hand, the emotional variables of adolescents of both sexes, in particular anger state, aggressive behaviour and empathic concern, and the unproductive coping and stress as a treat variables evaluated in time 2, the ones that stand out in terms of predictor power of anxiety in adolescents.

## Discussion and Conclusions

The results of the study contribute relevant information about the cognitive processes and the emotions to be taken into consideration to prevent anxiety, as well as the gender differences in the evaluated variables. Adolescent girls show more anxiety symptoms and more empathic concern throughout adolescence, while the boys show more emotional instability and more aggressive behaviour. In reference to the coping mechanism which adolescents use to solve problems or face situations that make them tense it is established that through adolescence these effective strategies for problem solving diminish, as they report that they have less mechanisms and resources oriented to problem solving, while at the same time they seek less support from others who could help them. Therefore, we can observe that, during the evaluated period, anxiety is particularly important in girls and emotional instability and aggressive behaviour in boys, while the mechanisms to cope or regulate said emotions diminish.

As to the psychological problems more related to anxiety and with a higher predictor power we can conclude that the emotions and the more immediate cognitive processes, meaning anger state, aggressive behaviour, empathic concern, unproductive coping mechanisms and the perception of stress as a threat evaluated the previous year weigh more heavily, while they don’t have the same influence over anxiety in the time 1 of the study.

Therefore, the development of coping mechanisms oriented to problem solving and to emotional self-control when facing situations that cause tension or conflicts that require a solution from the individual and impulsiveness control are processes that need to be taught and developed during adolescence to contribute to the reduction of anxiety, to a good emotional balance related to a more adapted behaviour (3,15).

To summarise, the results indicate that adolescence anxiety treatment or prevention programs should include the recognition and the acceptance of emotions, emotional self-regulation, as well as the acquisition of coping mechanisms to face situations that produce tension or are perceived as a threat to the adolescent.
